# Microstructure Evolution of Ni_3_Al-Based Intermetallic Alloy Strips After Hot Rolling

**DOI:** 10.3390/ma18133016

**Published:** 2025-06-25

**Authors:** Paweł Jóźwik, Wojciech Polkowski, Andrzej J. Panas, Zbigniew Bojar

**Affiliations:** 1 Faculty of Advanced Technologies and Chemistry, Institute of Materials Science and Engineering, Military University of Technology, Kaliskiego 2 Str., 00-908 Warsaw, Poland; zbigniew.bojar@wat.edu.pl; 2 Łukasiewicz Research Network—Krakow Institute of Technology, Zakopiańska 73 Str., 30-418 Krakow, Poland; wojciech.polkowski@kit.lukasiewicz.gov.pl; 3 Faculty of Mechatronics, Armament and Aerospace, Institute of Aerospace Technology, Military University of Technology, Kaliskiego 2 Str., 00-908 Warsaw, Poland; andrzej.panas@wat.edu.pl

**Keywords:** Ni_3_Al-based intermetallic alloy, hot rolling, dynamic recrystallization, EBSD analysis, heat conduction model

## Abstract

The effect of the temperature and strain rate during the hot rolling process on the microstructural evolution in fine-grained Ni_3_Al intermetallic alloy doped with Zr and B was examined in this work. The hot rolling process was carried out at an initial temperature range of 1000, 1100, and 1280 °C and at a strain rate between 3.9 × 10^−1^ s^−^^1^ and 2.5 s^−^^1^. The results of the EBSD microstructural analyses revealed that dynamic recrystallization phenomena are initiated at the rolling temperature of 1100 °C, while a fraction of the dynamically recrystallized grains further increases with both the rising temperature and strain rate of the deformation process. Furthermore, to estimate the heat losses during the hot rolling processing, a non-stationary heat transfer model was formulated and then used to evaluate the experimentally received data.

## 1. Introduction

Ni_3_Al-based intermetallic alloys demonstrate superior characteristics compared with conventional heat-resistant nickel-based superalloys, including significantly higher strength at elevated temperatures, relatively lower density, and improved resistance to corrosive environments. An analysis of the properties of these materials and their commercialization potential has been extensively presented by Sikka et al. [1,2], Jozwik et al., [3], Jarosz et al. [4], Sahare et al. [5], Sun and Yuan [6], and Tiwari et al. [7]. Their findings indicate that Ni_3_Al intermetallics in bulk form are predominantly utilized in the fabrication of dies and press tool components, equipment for furnaces used in thermochemical treatment, and jet engine components. Considering the aforementioned unique physical and chemical properties of these materials, their potential applications in the form of strips or foils in various advanced components, including electromechanical systems (such as heat exchangers, reactors, actuators, and glucose sensors), solar cells, automotive and aircraft engine components, and thermocatalytic systems, have been proposed.

One possible approach to obtain Ni_3_Al strips and foils with enhanced strength and ductility is hot plastic processing. Notably, the literature contains limited information regarding the hot-working of Ni_3_Al-based intermetallic alloys. Furthermore, the available studies are limited to only a few research examples related to the hot processing of bulk materials (i.e., samples with a dimension of at least 8 mm). The studies under consideration include isothermal compression tests (Wang et al. [8], Wu et al. [9], Zhong et al. [10,11], Prasad et al. [12]); hot extrusion (Guo et al. [13], Sheng et al. [14]); compression tests with heated swages; and rotary working tests with localized heating evoked by friction (Schindler et al. [15,16]). The results mentioned in these studies indicate that appropriately selected parameters of the deformation process (temperature, strain rate and degree of deformation, state of the initial structure, and a presence of alloying elements) lead to microstructural changes in Ni_3_Al-based alloys as a result of dynamic recrystallization (DRX) or post-dynamic recrystallization. As observed by Bhatia [17] and Ponge et al. [18], the DRX of Ni_3_Al-based alloys can result in the presence of a “necklace structure” characterized by fine recrystallized grains surrounding large primary grains of γ’ phase. Such a structure can play a pivotal role in achieving the desired combination of high creep and low cycle fatigue resistance. Despite these advances, the impact of specific hot deformation parameters on the microstructural evolution and mechanical properties of Ni_3_Al-based alloys remains insufficiently understood, particularly for materials in the form of thin strips or foils. It is worth noting that the hot rolling process used in the presented work has many varieties described in the literature, including rotational hot rolling (RHR) [19], asymmetric rolling (ASR) [20], radial shear rolling (RSR) [21], corrugated rolling [22], etc.

The main objective of this study was to analyze the DRX-induced microstructural changes in Ni_3_Al-based intermetallic strips. Because of the significantly limited dimensions (thickness of approximately 1 mm), it should be assumed that the proposed hot plastic processing will occur under conditions of intensive heat exchange between the treated material strips and the working tools (rollers).

An additional aspect of this study is to explore the possibility of obtaining transient strengthening states of Ni_3_Al-based alloys with the level of mechanical properties controlled by the occurrence of the necklace structure obtained by hot rolling.

## 2. Experimental Procedure

The hot rolling process was performed on Ni_3_Al-based intermetallic alloy strips with a chemical composition of Ni–22.1Al–0.26Zr–0.1B (at.%), using rolling mill sexto. The material in the initial state (a strip of 150 mm × 30 mm × 1.12 mm) was obtained by consecutive cold rolling and heat treatment (with strictly selected conditions of deformation and recrystallization) to obtain a single-phase fine-grained structure (grain size of d~30 µm) of the γ’ phase. Details of the Ni_3_Al strip production process were reported previously by Jóźwik et al. [23,24,25]. The obtained material constituted the “initial” state for the hot rolling. The processing was carried out by pre-heating the Ni_3_Al-based strips to 1000 °C, 1100 °C, or 1280 °C. Various strain rate $\dot{\varepsilon }$ values were applied: 3.9× 10^−^^1^ s^−^^1^, 9.9 × 10^−1^ s^−^^1^, 1.5 s^−1^, or 2.5 s^−1^ (the last one was not used for T = 1280 °C). For all hot rolling conditions, the total strain value (i.e., expressed as a relative thickness reduction) was 0.3, and it was set in one pass. To eliminate the possibility of post-dynamic recrystallization in the material, after each hot rolling operation, the samples were immediately (in less than 1 s) cooled in a water bath. Then, an electric discharge machining was used to cut the obtained strips in a plane perpendicular to the rolling direction located in the middle of the length of the rolled material. Metallographic cuts were ground on SiC papers and mechanically polished using diamond suspensions of 3 µm and SiO_2_ suspension of 0.1 µm. Microstructural studies were performed by using a Quanta 3D FEG (Thermo Fisher Scientific, Waltham, MA, USA) scanning electron microscope (SEM) equipped with an electron backscattered diffraction detector (EBSD) (Ametek, Berwyn, PA, USA) at an accelerating voltage of 20 kV, a beam current of 8 nA, and analysis step (depending on magnification) from 1 µm to 0.1 µm.

Based on the results of the systematic EBSD mapping of crystallographic orientations, the grain structure of the polycrystalline strips was reconstructed, revealing positions of high- and low-angle grain boundaries, simultaneously. In turn, the maps of the surface distribution of the poles of the mean crystallographic orientation of individual grains inverse pole figure (IPF) maps were the basis for assessing the presence of privileged crystallographic orientation and possible micro-texture. The analysis of structural changes as a result of the performed hot rolling procedures was based on the measurements of the degree of local disorientation of the crystal lattice in the volume of individual grains, presented in the form of grain orientation spread (GOS) parameter maps. In this study, the average GOS parameter, which shows the average value of the disorientation of the particular investigated area, was used. The analysis of the quality of the Kikuchi lines, thereby determining the image quality (IQ) parameter, was also performed. The primary source of imperfections in the measurements of the GOS and IQ parameters is the dependence of the test results on factors related to both the measurement parameters used and the quality of preparation. Therefore, during the sample preparation and testing, special attention was paid to maintaining the repeatability of the applied experimental conditions [26,27,28,29,30].

Finally, hardness measurements were performed in the rolling plane using the Vickers method (HV5), with a load of 5 kg and a 10 s loading time in single measurement (according to the ISO 6507-1:2023 standard [31]), and the average value and standard deviation were determined based on at least ten measurements for each tested material variant.

Additionally, to estimate the temperature changes in the Ni_3_Al strips during hot rolling, a model of non-stationary heat conduction in a hot-rolled metal plate was formulated using the Comsol/FEM (finite element method) ver. 6.3 (Comsol, Burlington, MA, USA) calculation package (more details are shown elsewhere [32]).

## 3. Results and Discussion

**Figure 1:**
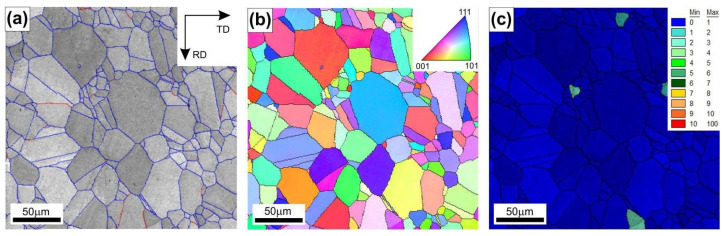
EBSD maps of Ni_3_Al-based intermetallic alloy in the initial stage: (**a**) reconstruction of high angle boundaries (blue color) and low angle boundaries (red color), (**b**) IPF map with high angle boundaries (≥15°) marked in black, and (**c**) internal grain orientation spread (GOS).

The examined Ni_3_Al-based intermetallic alloy in the initial stage (before the hot rolling) exhibited a single-phase fine-grained γ’ secondary solid solution structure (Figure 1). The results of the EBSD analyses revealed the following features: (i) a dominant (approximately 95%) fraction of high angle grain boundaries (with a disorientation angle greater than 15° between neighboring grains) (Figure 1a), (ii) no visible crystallographic texture (Figure 1b), and (iii) a very low value of the GOS parameter (internal grain orientation spread ≤ 1°), which is specific for a fully recrystallized material (Figure 1c).

The GOS parameter (expressed in angular degrees) measures the scattering of the crystal lattice misorientation inside an individual grain. This parameter assumes that plastic deformation in a metallic polycrystalline material is assisted by local changes in the crystal lattice orientation in the volume of each individual grain. This crystal lattice evolution is necessary to maintain the cohesion along the grain boundaries of a material subjected to a plastic deformation. For thermally activated processes (i.e., recovery and recrystallization), this parameter enables a quantitative assessment of the extent of these processes, as presented by Schwartzer et al. [33], Conde et al. [34], and Esin et al. [35]. In a polycrystalline structure, grains with a very low fraction of structural defects (typically after the recrystallization process, as in the case of the analyzed alloy in the initial state) show negligible differentiation of local crystallographic orientations, and the value of the GOS parameter is ≤1°. In turn, deformed grains with a high density of crystal defects show a high value of internal misorientation and, therefore, a high value (GOS > 1°) of this parameter.

The IQ parameter expresses a strong relationship between the degree of crystal structure defects (e.g., as a result of plastic deformation) and the quality (blurring) of the backscattered electron diffraction lines. The electron beam interacting with the surface layer of the sample is dispersed on the crystal lattice’s defects, and the crystal planes’ curvatures are “bent” due to plastic deformation. Consequently, the diffraction lines obtained from the subareas with a high density of lattice defects are blurred and visible as dark. According to Ku [26] and Alam et al. [36], IQ values can be correlated with the dislocation density, thereby providing a means of quantifying the internal strain energy and residual stress within a material. This correlation can be particularly valuable for understanding the effects of thermal treatments and mechanical processing on material properties [37].

**Figure 2:**
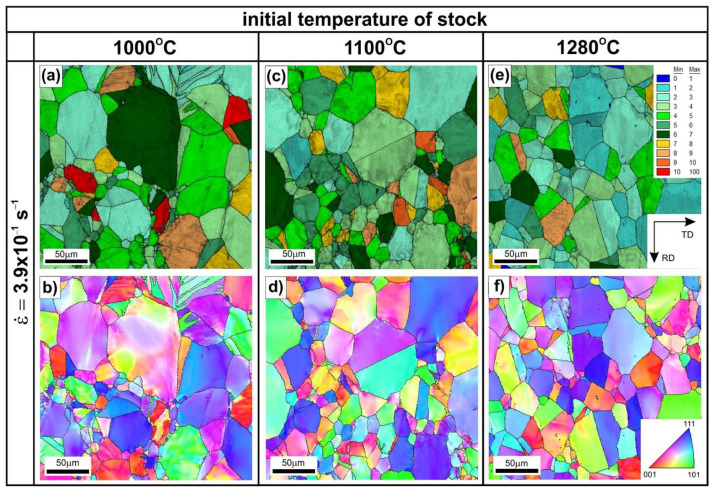
The grain orientation spread (GOS) maps (**a**,**c**,**e**) and IPF maps of with high angle boundaries (≥15°) marked by black lines (**b**,**d**,**f**) of Ni_3_Al-based strips after hot rolling up to 0.3 strain, at a strain rate $\dot{\varepsilon }$ = 3.9 × 10^−1^ s^−1^, at the initial temperatures of (**a**,**b**) 1000 °C, (**c**,**d**) 1100 °C, and (**e**,**f**) 1280 °C.

**Figure 3:**
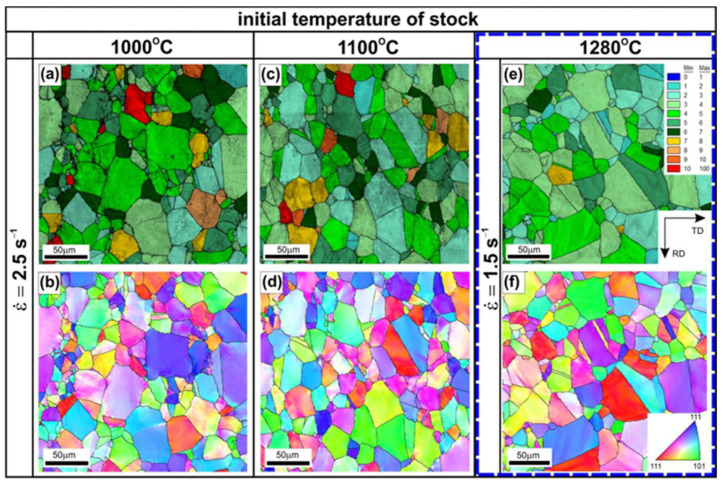
The grain orientation spread (GOS) maps (**a**,**c**,**e**) and the IPF maps with high angle boundaries (≥15°) marked by black lines (**b**,**d**,**f**) of Ni_3_Al-based strips after hot rolling up to 0.3 strain, at a strain rate of $\dot{\varepsilon }$ = 2.5 s^−1^, at the initial temperatures of (**a**,**b**) 1000 °C and (**c**,**d**) 1100 °C, and at a strain rate of $\dot{\varepsilon }$ = 1.5 s^−1^ at a temperature of 1280 °C—(**e**,**f**).

**Figure 4:**
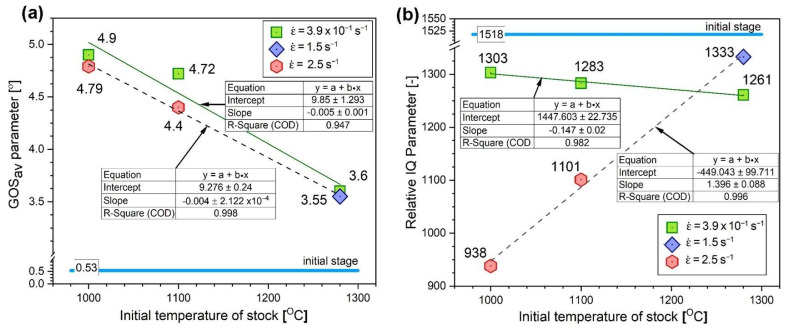
The results of quantitative EBSD analyses obtained for the investigated material: (**a**) average GOS values and (**b**) relative IQ values.

**Figure 5:**
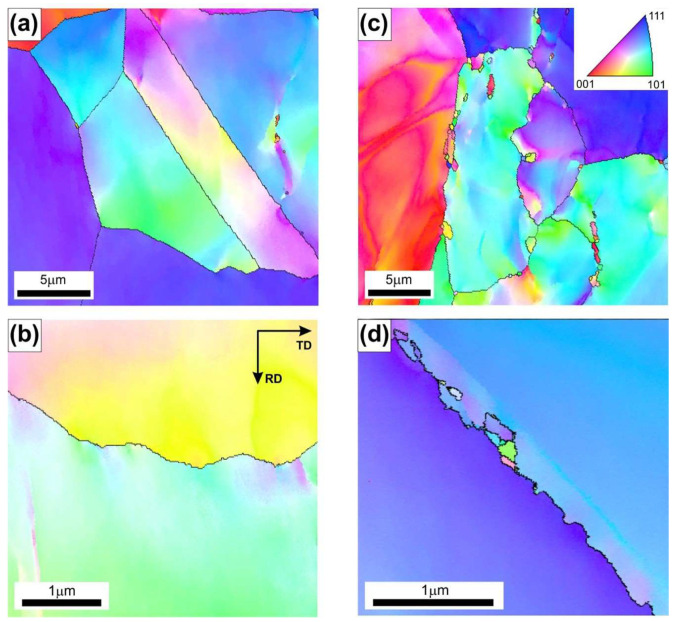
The EBSD IPF maps with high angle boundaries marked by black lines of Ni_3_Al-based intermetallic alloy after hot rolling at T = 1000 °C at $\dot{\varepsilon }$ of (**a**,**b**) 3.9 × 10^−1^ s^−1^ and (**c**,**d**) 2.5 s^−1^.

**Figure 6:**
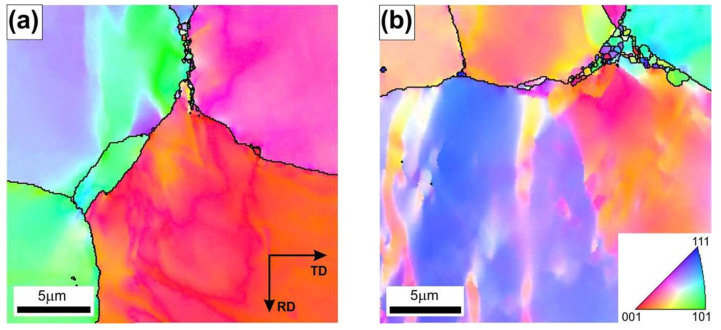
The EBSD IPF maps with high angle boundaries marked by black lines of Ni_3_Al-based intermetallic alloy after hot rolling at T = 1100 °C and $\dot{\varepsilon }$ of (**a**) 3.9 × 10^−1^ s^−1^ and (**b**) 2.5 s^−1^.

**Figure 7:**
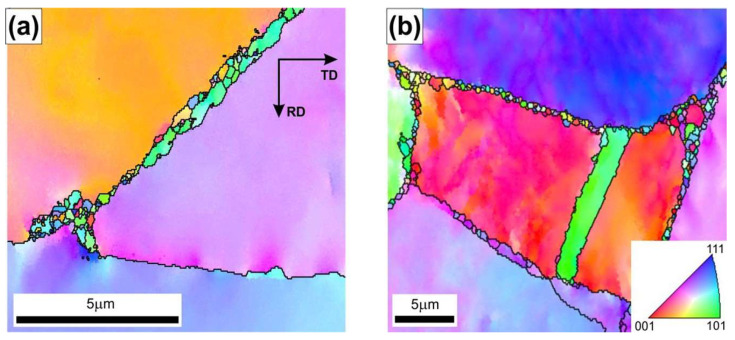
The EBSD IPF maps (high angle boundaries marked by the black lines) of Ni_3_Al-based alloy after hot rolling at T = 1280 °C and at $\dot{\varepsilon }$ of (**a**) 3.9 × 10^−1^ s^−1^ and (**b**) 1.5 s^−1^.

**Figure 8:**
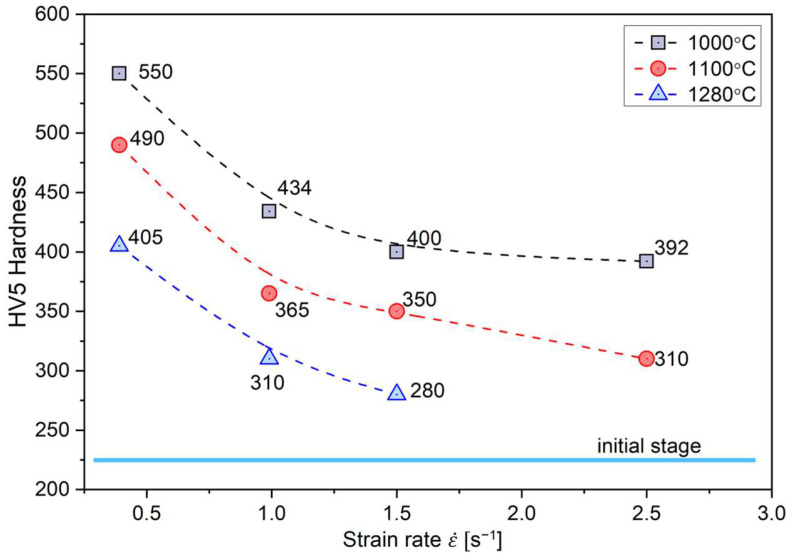
Influence of strain rate $\dot{\varepsilon }$ and initial temperature of stock on Vickers hardness of Ni_3_Al-based strips (average values).

Hot rolling of the Ni_3_Al-based alloy to a strain value of 0.3 has a noticeable effect on both the microstructure (Figure 2, Figure 3, Figure 4, Figure 5, Figure 6 and Figure 7) and hardness (Figure 8) of the tested material, depending on the applied temperature and the strain rate. The results of EBSD analyses (compare Figure 1, Figure 2 and Figure 3) showed a limited extent of structural reconstruction. A comparative assessment of the GOS parameter for the material after the hot rolling showed a noticeable increase in its value compared to the starting material (the non-deformed one), with the presence of grains exhibiting a GOS >10° (compare Figure 1c, Figure 2a,c,e, and Figure 3a,c,e).

The quantitative evaluation of the averaged GOS parameter values clearly shows the impact of the rolling parameters on the extent of the structural reconstruction (Figure 4a). A much more significant influence of the deformation temperature was observed in relation to the strain rate. For the hot rolling process carried out at the high strain rate ($\dot{\varepsilon }$ ≥ 1.5 s^−1^), a similar tendency was observed. An almost linear dependence of the average GOS value on the temperature was obtained (Figure 4a). The influence of a high strain rate on the course of DRX is also indicated by the analysis of the IQ parameter—for the value $\dot{\varepsilon }$ ≥ 1.5 s^−1^—increased almost linearly with the deformation temperature (Figure 4b). The IQ parameter value did not show a significant dependence on the temperature due to the hot rolling at a strain rate $\dot{\varepsilon }$ of 3.9 × 10^−1^ s^−1^, and the observed changes were insignificant (approximately 3%).

The results of more detailed EBSD analyses carried out by using higher magnifications revealed the locally occurring effects of thermally activated processes with intensity dependent on the values of the applied processing parameters (Figure 5, Figure 6 and Figure 7). The material deformed at T = 1000 °C, with a strain rate of 3.9 × 10^−1^ s^−1^ (Figure 5a,b), did not show the presence of structural features that are typical for the DRX.

Increasing the strain rate at this temperature resulted in a growing fraction of very fine DRX grains (Figure 5). The serration of primary grain boundaries was observed (Figure 5c,d) in the sample hot-rolled at T = 1000 °C and at a strain rate of 2.5 s^−1^. Analogous observations were reported by Ponge et al. [18], Sakai et al. [38], and Koundinya et al. [39], according to whom such structural features are specific to a deformation temperature lower than the critical temperature for initiating the dynamic recrystallization process. The serration of grain primary boundaries was also observed after the hot rolling at T = 1100 °C and at a strain rate of 9.9 × 10^−1^ s^−1^.

Therefore, an increase in the initial strip temperature (up to 1100 °C) promotes the formation of new grains by the DRX in sites with the highest stress concentration, i.e., the triple-junctions of grain boundaries (Figure 6), as well as along the straight segments of primary grain boundaries. The share of grain boundary segments affected by the DRX increased with an increase in the strain rate, which was also reflected in the observed decrease in the strain strengthening (Figure 8).

After the hot rolling at T = 1280 °C, the material showed the largest share of DRXed grains both in the triple-junction grain boundaries and along the primary grain boundaries (Figure 7b). As reported by Bhatia et al. [17], the tendency to form necklace structures in the Ni_3_Al-based alloys is caused by the tendency of planar slip and difficulty of cell formation inside the grains. Karnthaler et al. [40], Kruml et al. [41], and Wang et al. [42] have classified the Ni_3_Al-based alloys as high stacking fault energy (SFE of approximately 240 mJ/m^2^) materials. However, the results of studies by Jozwik et al. [23,25] and Polkowski et al. [43] indicate that the behavior of materials from this group under plastic deformation conditions and subsequent thermally activated processes is more similar to that of low SFE materials, as they are characterized by intensive strain hardening (resulting from hindered transverse slip of screw dislocations and a tendency to form flat stacking of dislocations on obstacles). Furthermore, as shown by Schindler et al. [15,16], the low SFE of Ni_3_Al-based alloys decreases susceptibility to the DRX. Consequently, a higher strain concentration at grain boundaries is obtained leading to the DRX in the necklace structure. Such a microstructure rebuilding, which is very similar to the model presented by Ponge et al. [18], was observed in the material after the hot rolling at a strain rate $\dot{\varepsilon }$ = 1.5 s^−1^ (Figure 7b).

Regardless of the applied hot rolling process parameters, the deformed material showed a slight increase in hardness as compared with the initial material, resulting from the increased stored deformation energy (Figure 8). Regardless of the applied hot rolling temperature, the increase in the strain rate resulted in a decrease in the strain hardening of the material. It was mostly visible in the case of the process conducted at the highest strain rate (i.e., 2.5 s^−1^). The obtained results correspond to the discussed changes in the EBSD examined microstructure (Figure 2, Figure 3, Figure 4, Figure 5, Figure 6 and Figure 7). In other words, the microstructural evolution is dominated by a dynamic recovery process with the DRX limited to some micro-areas. As the dynamic recovery creates low-energy dislocation systems (polygonal walls and subgrains), it effectively contributes to a reduction in the total energy stored.

The obtained results show that under the applied experimental conditions, a higher strain rate favors the microstructure evolution by both the DRX and dynamic recovery. This unusual relationship is probably due to the very low heat storage of the rolled Ni_3_Al strips; that is, the initial thickness was 1.12 mm (for comparison, the smallest dimension of the Ni_3_Al samples analyzed in the literature is 8 mm), which, in combination with cold work rolls, resulted in greater heat losses, leading to a significant reduction in the actual material temperature during the deformation process. Consequently, a higher strain rate corresponded to a relatively higher process temperature.

To estimate the possible temperature changes in the Ni_3_Al strips during the hot rolling, as well as to verify the obtained research results, a non-stationary model of heat conduction in a hot-rolled metal plate was formulated.

Regardless of the applied hot rolling process parameters, the deformed material showed a significant increase in hardness as compared with the initial material, resulting from the increased stored deformation energy (Figure 8). Regardless of the applied hot rolling temperature, the increase in the strain rate resulted in a decrease in the strain hardening of the material. It was mostly visible in the case of the process conducted at the highest strain rate (i.e., 2.5 s^−1^). The obtained results correspond to the discussed changes in the EBSD examined microstructure (Figure 2, Figure 3, Figure 4, Figure 5, Figure 6 and Figure 7). In other words, the microstructural evolution is dominated by a dynamic recovery process with the DRX limited to some micro-areas. As the dynamic recovery creates low-energy dislocation systems (polygonal walls and subgrains), it effectively contributes to a reduction in the total energy stored.

### 3.1. A Non-Stationary Heat Conduction Model in a Strip

**Figure 9:**
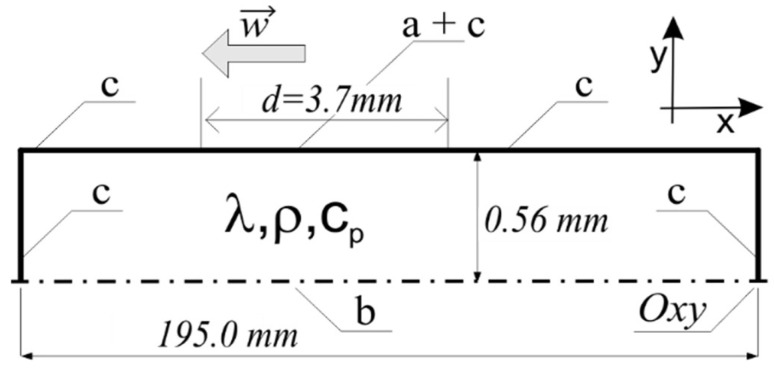
Schematic diagram of the numerical model of heat conduction in a rolled plate (description in the text).

The primary purpose of the modeling was to qualitatively determine the temperature changes. For this purpose, the issue was simplified to a model of a heat source moving across the surface of the component. Thus, a contact surface having a linear dimension d was set as the source of heat and, at the same time, the heat receiver (Figure 9).

To determine the temperature changes and strain distribution, the presence of the following phenomena were analyzed [44]: (i) the heat conduction in the workpiece to be deformed (i.e., the Ni_3_Al strip); (ii) convective and radiative heat losses from the workpiece surface to the environment; (iii) the heat conduction in the machining tool (roller); (iv) the contact heat transfer resistance between the workpiece and the tool; (v) the friction between the workpiece and the roller surface contributing to the surface heat source; (vi) the elastic deformation of the workpiece; (vii) the plastic deformation constituting volumetric heat sources, (viii) the elastic relaxation of the material as it comes off the roller; and (ix) the change in the relative velocity of movement of the workpiece and the roller due to deformation. In the presented simplified model of a moving heat source, only phenomena (i)–(iv) were directly taken into account. A phenomenon that is usually overlooked at this stage of simplification is the volumetric shrinkage caused by thermal expansion.

The relatively large value of the ratio of the wafer width to its thickness provides a justification for adopting a simplified two-dimensional heat conduction model. A geometrical scheme of the model is illustrated in Figure 9.

Thus, the issue was reduced to a two-dimensional model of a non-stationary heat transfer in a plate with dimensions of 195 mm × 0.56 mm, subjected to a surface moving heat source (surface a in Figure 9) [45]. A symmetric condition in the plane containing the Ox axis was assumed (surface b in Figure 9), while the convection and radiation losses were considered for other surfaces (surface c in Figure 9). Due to the lack of data on the constitutive model, the volumetric heat sources accompanying plastic deformation, as well as the surface frictional heat source, were omitted. An evaluation of the literature data shows that despite the noticeable impact of these effects, they do not play a dominant role in shaping the temperature distribution at a relatively high initial temperature of the hot-machined workpiece (i.e., Ni_3_Al strip).

The mathematical equations describing the considered model (in the Oxy coordinate system, Figure 9), are as follows:

- equation for transient heat conduction:

(1) $$\rho c_{p}\frac{𝜕T}{𝜕\tau }+\nabla \left(-\lambda \nabla T\right)=0;$$

- initial homogeneous condition:

(2) $$T\left(x,y,\tau =0\right)=T_{0};$$

- boundary condition for the adiabatization of the horizontal center symmetry surface of the plate (surface marked as “b” in Figure 9):

(3) $${\left.\frac{𝜕T}{𝜕y}\right|}_{S}=0;$$

- boundary conditions of convective and radiative heat loss (surfaces marked “c” in Figure 9, “n” is a normal direction to surfaces c):

(4) $$-\lambda {\left.\frac{𝜕T}{𝜕n}\right|}_{S}=\alpha \cdot \left({\left.T\right|}_{S}-T_{amb}\right)+\varepsilon \;\sigma \;\left[{\left({\left.T\right|}_{S}\right)}^{4}-{T_{amb}}^{4}\right];$$

- the displacement contact zone is modeled by the additional contact loss (surface marked as “a + c” in Figure 9) of the plate surface with the surface of the cylinder at ambient temperature, with the value of the thermal contact resistance *r_cont_*:

(5) $$-\lambda {\left.\frac{𝜕T}{𝜕y}\right|}_{S}=h_{r}\cdot \left({\left.T\right|}_{S}-T_{amb}\right)=\frac{1}{r_{cont}}\cdot \left({\left.T\right|}_{S}-T_{amb}\right)\;\mathrm{for}-w\cdot \tau <x<-w\cdot \tau +d$$

The last boundary condition was applied in superposition with Equation (4). The equation describes contact with the cylinder surface excluding radiation losses and convective losses. On the other hand, in the considered case, these losses account for only approximately 2% of the losses caused by a heat transfer through the contact zone. Because determination of the value of thermal contact resistance is subjected to a larger error and because of the need for computational optimization of the numerical model, the arrangement of conditions (4) and (5) remained unchanged.

### 3.2. Numerical Model

The tools of the Comsol/M FEA (finite element method) calculation package were used to build the numerical model. The temperature dependence of the material parameters of the analyzed alloy was considered (Table 1) by extrapolating data from the studies by Panas et al. [46] and Terpilowski [47]. Table 2 presents the values of the parameters defining the boundary conditions developed based on the analysis of the criterion relations of the heat transfer issue and the data contained in the aforementioned monograph by Lenard et al. [43]. Based on these data, the value of the equivalent heat transfer coefficient in the contact resistance zone was assumed to be 13,000 W × m^−2^ × K^−1^, which corresponds to the value of *r_cont_* given in Table 2.

The analysis considered the realistic conditions of the rolling process, including the thermal exchange between the sample and its environment during the 1 s period from when the sample was removed from the furnace to when it came into contact with the rollers. Consequently, the initial temperature of the stock was reduced, i.e., from 1280 °C of the initial temperature of stock to 1210 °C, from 1100 °C to 1056 °C, and from 1000 °C to 967 °C. Regular rectangular finite elements were used for calculations. A division of eight elements was used in the vertical direction, and 780 elements were used in the horizontal direction. The horizontal division density was set as 1 mm. Numerical simulations of the heat conduction process were performed with a constant time step. For the highest strain rate (i.e., 2.5 s^−1^), the time step was 0.0015 s, and for the lowest (i.e., 3.9 × 10^−1^ s^−1^), it was 0.0075 s.

**Figure 10:**
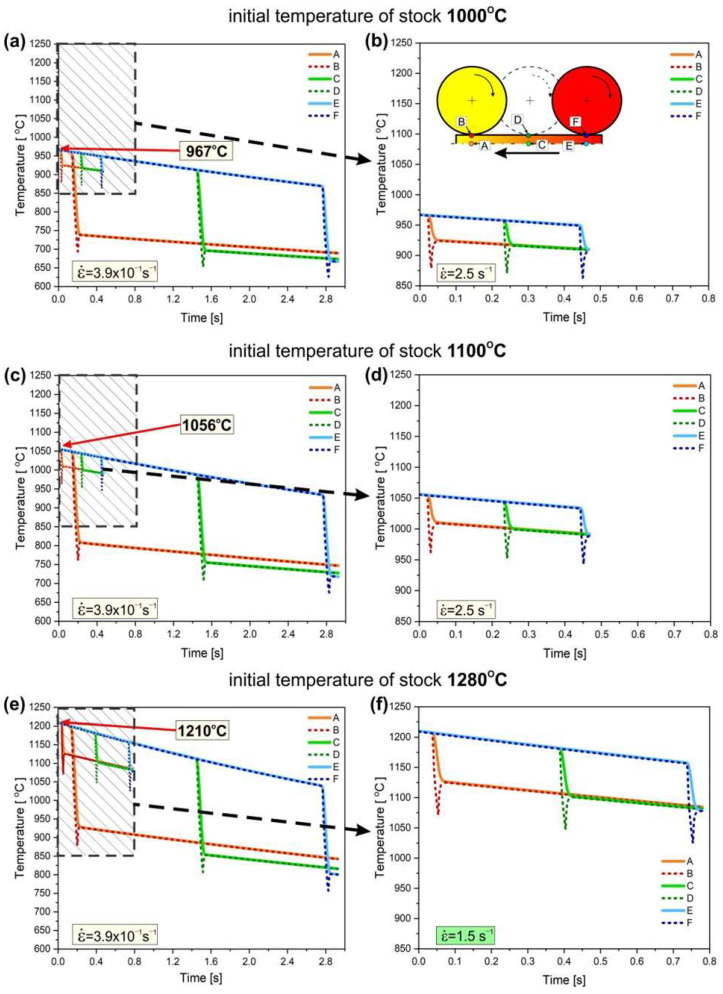
The course of temperature changes obtained from the numerical model at individual points of the rolled strip during hot rolling ((**a**,**c**,**e**)—core, (**b**,**d**,**f**)—surface).

To visualize the obtained calculation results, control points were defined at the rolled band side surface and core (Figure 10):

- In the plane perpendicular to the rolling direction, core (points A, C, and E) and contact surface of the rollers (points B, D, and F).
- In the plane parallel to the rolling direction, the beginning (points A and B), center (points C and D), and the end of the sample (points E and F).

In terms of the 2D nature of the model, the points represent lines parallel to the roller axis. In the model, it was assumed that points A and B were located 10 mm from the start of the sample, points C and D were positioned at the center of the sample, and points E and F were placed 10 mm before the sample’s end.

The results of the modeling showed a significant effect of the strain rate on the change in the material temperature during the rolling process. As expected, the most significant changes in each of the analyzed cases were observed at the surface of the sample directly in contact with the rollers (points: B, D, and F, respectively). After an initial decrease in the temperature of the band, a slight increase was observed because of the heat transmission from the core of the material and the heating of the rollers. Owing to its higher heat capacity, the core point (i.e., points A, C, and E) turned out to be slightly more temperature stable, and the observed changes occurred with significantly lower intensity (Figure 10).

The greatest cooling of the rolled band was observed for the highest initial temperature of stock, i.e., 1280 °C (reduced before contact with the rollers to 1210 °C as a consequence of the heat exchange with the environment) and the lowest strain rate (i.e., $\dot{\varepsilon }$ = 3.9 × 10^−1^ s^−1^). Under these conditions, the temperature gradient was the most significant, and the contact time between the rollers and the workpiece material was the longest. Consequently, the temperature in the core of the material decreased by approximately 280 °C at the beginning and 410 °C at the end of the sample. Notably, despite the largest temperature change among the analyzed cases, its value remained above 800 °C throughout the deformation process. The increase in the strain rate for this processing temperature resulted in a significantly higher value of the temperature during deformation (i.e., above 1070 °C for $\dot{\varepsilon }$ = 1.5 s^−1^) owing to the shorter contact time of the rollers with the material. The analyzed temperature changes correspond with the observed microstructural restoration by dynamic recrystallization, the contribution of which increases with the increasing strain rate (Figure 7).

At the initial temperature of the stock to 1100 °C, which was reduced to 1056 °C by losses related to heat exchange with the environment, and the strain rate $\dot{\varepsilon }$ = 3.9 × 10^−1^ s^−1^, there was less cooling of the material, by approximately 250 °C at the beginning and 340 °C at the end of rolling, respectively. However, it is worth noting that, in this case, the temperature decreased to approximately 800 °C at the beginning and 720 °C at the end of the band. As shown by the studies of Prasad et al. [12], Schindler et al. [15,16], and Jóźwik et al. [19,24], this value was not sufficient to initiate recrystallization processes. Consequently, for these rolling conditions (i.e., 1100 °C of the stock and $\dot{\varepsilon }$ = 3.9 × 10^−1^ s^−1^), only a slight visible rebuilding effect in the microstructure by dynamic recrystallization was observed (Figure 6), with a simultaneous reduction in the strengthening of the materials under these conditions (Figure 8). Increasing the strain rate $\dot{\varepsilon }$ to 2.5 s^−1^, with an initial stock temperature of 1100 °C, resulted in a reduced heat transfer with the rollers and consequently a higher material temperature in the core during the deformation process, which was approximately 1010 °C at the beginning and 990 °C at the end of the band. As with the deformation at highest initial temperature of stock (i.e., 1280 °C), dynamic recrystallization processes are most evident for the highest strain rate, which provides the shortest contact time between the rollers and the material (Figure 4).

A further decrease in the temperature of the stock down to 1000 °C (reduced to 967 °C by losses related to heat exchange with the environment) and the strain rate $\dot{\varepsilon }$ = 3.9 × 10^−1^ s^−1^ resulted in a temperature decrease to approximately 740 °C at the beginning and 670 °C at the end of the band. As previously mentioned, this temperature was definitely too low to initiate the dynamic recrystallization process. As a result, no visible effects of the microstructure evolution by dynamic recrystallization were observed under these rolling conditions (Figure 5). Simultaneously, the level of strengthening was the highest among all the analyzed process conditions (Figure 8). Increasing the strain rate $\dot{\varepsilon }$ to 2.5 s^−1^, with a constant temperature of stock (i.e., 1000 °C), resulted in a higher material temperature in the core during the deformation process: 925 °C at the beginning and 910 °C at the end of the band.

Similar to the deformation processes realized at 1280 °C and 1100 °C, the dynamic recrystallization process was mostly observed for the material processed at the highest strain rate $\dot{\varepsilon }$ because of the lower undercooling and higher rolling temperature (Figure 10).

**Figure 11:**
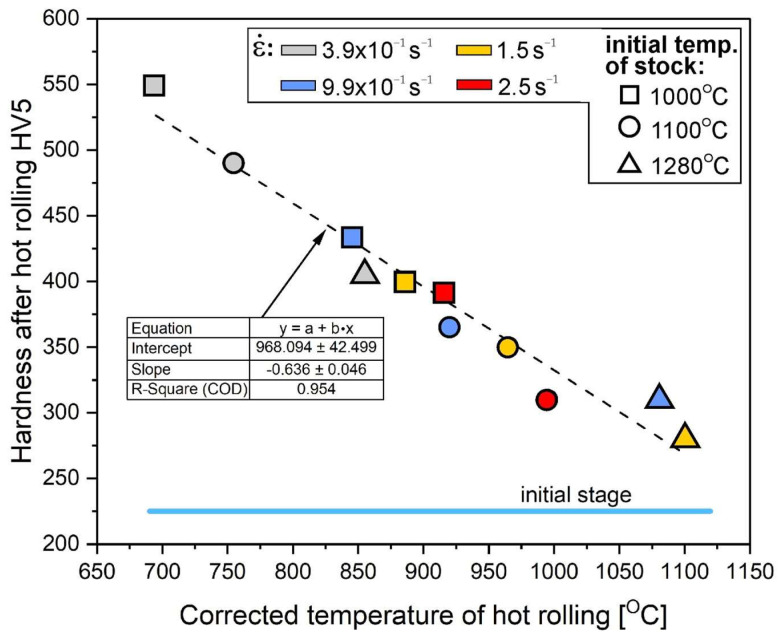
Effect of the numerical modeling-determined temperature in the core of Ni_3_Al strips’ center (Figure 10, point C) during rolling on HV5 hardness, taking into account the rolling process parameters (average values).

Finally, considering the corrected temperature in the core of the material (obtained from the model, Figure 10, point C), an almost linear effect on strengthening was observed (Figure 11). It is a macroscopic determinant of the intensity of the heat-activated processes of the structure restoration (recovery and recrystallization), which consequently fully correspond to the analysis of EBSD results presented above (Figure 4, Figure 5 and Figure 6).

## 4. Summary and Conclusions

The conditions of the hot rolling process applied in this study noticeably affect the microstructure of Ni_3_Al strips by inducing local dynamic recrystallization phenomena. Simultaneously, the hot deformation processing induces an intensive increase in strengthening. The observed behavior suggests the potential for hybrid process development, which warrants further investigation. Such an increase in strength suggests that a significant part of the strain energy was stored in the material during hot forming, which demonstrates the limited extent of the structural evolution through dynamic restoration processes (recovery and recrystallization). The unusual effect of the strain rate on the aforementioned microstructure rebuilding processes was verified using a model of non-stationary heat conduction. Based on the obtained modeling results, a significant effect of the strain rate on the temperature of the material during rolling was found. The low heat capacity of Ni_3_Al strips with an initial thickness of 1.12 mm combined with cold working rolls, caused a significant reduction in the actual temperature of the deformation process. Consequently, a higher strain rate $\dot{\varepsilon }$ corresponds to a higher material temperature during deformation.

Considering some limitations of the model adopted in this study (i.e., no in situ temperature measurement of the material temperature during deformation, the use of estimated thermal contact resistance values, and the assumption of uniform initial conditions across the strip), the authors plan to verify them in real conditions.

## Figures and Tables

**Table 1:** Material properties adopted for the calculations based on extrapolation of the results of measurements by Panas [46] and Terpilowski [47] for the Ni-22.1Al-0.26Zr-0.1B (at.%) alloy.

Temperature	Specific Heat	Relative Elongations	Density	Thermal Diffusivity	Thermal Conductivity
*t* [°C]	*c_p_* [J·kg^−1^·K^−1^]	*ε* [mm·m^−1^]	*ρ* [kg·m^−3^]	*a* [mm^2^·s^−1^]	*λ* [W·m^−1^·K^−1^]
0	466	−0.31	7592	4.27	15.1
100	482	0.91	7564	4.75	17.3
200	499	2.21	7535	5.24	19.7
300	515	3.58	7504	5.73	22.1
400	532	5.02	7472	6.21	24.7
500	548	6.54	7438	6.70	27.3
600	564	8.13	7403	7.18	30.0
700	581	9.80	7366	7.67	32.8
800	597	11.54	7328	8.16	35.7
900	614	13.35	7289	8.64	38.7
1000	630	15.23	7249	9.13	41.7
1100	647	17.19	7207	9.61	44.8
1200	663	19.22	7164	10.10	48.0
1300	680	21.33	7120	10.59	51.2

**Table 2:** The values of parameters characterizing the initial and boundary conditions adopted for the calculations.

Temperature		Heat Transfer Coefficient	Thermal Contact Surface Resistance	Surface Emissivity
*t* [°C]	*T* [K]	*α* [W·m^−2^·K^−1^]	*r_cont_* [m^2^·K·W^−1^]	*ε* [-]
20	293	10	7.69 × 10^−5^	0.7

## Data Availability

The data presented in this study are available on request from the corresponding author due to privacy.

## Abbreviations

The following abbreviations are used in this manuscript:

$\dot{\varepsilon }$	Strain rate
DRX	Dynamic recrystallization
EBSD	Backscattered diffraction detector
IPF	Inverse pole figure
GOS	Grain orientation spread parameter
IQ	Image quality parameter
SFE	Stacking fault energy
*ρ*	Density
*c_p_*	Specific heat
*T*	Temperature in Kelvin
*t*	Temperature in °C
*T_amb_*	Ambient temperature
*τ*	Time
*λ*	Thermal conductivity
*x*	Horizontal coordinate
*y*	Vertical coordinate
*α*	Heat transfer coefficient
*ε*	Surface emissivity
*σ*	Stefan–Boltzmann constant
*h_r_*	Thermal contact heat transfer coefficient
*r_cont_*	Thermal contact surface resistance
